# Improving Visible Light Photocatalysis Using Optical Defects in CoO_x_-TiO_2_ Photonic Crystals

**DOI:** 10.3390/ma17235996

**Published:** 2024-12-07

**Authors:** Alexia Toumazatou, Elias Sakellis, Vlassis Likodimos

**Affiliations:** 1 Section of Condensed Matter Physics, Department of Physics, National and Kapodistrian University of Athens, University Campus, 15784 Athens, Greece; alextoum@phys.uoa.gr (A.T.); e_sakel@phys.uoa.gr (E.S.); 2 Institute of Nanoscience and Nanotechnology, National Center for Scientific Research “Demokritos”, Agia Paraskevi, 15341 Athens, Greece

**Keywords:** photonic crystals, titanium dioxide, cobalt oxides, inverse opals, photocatalysis, visible light, optical defects, slow photons, multilayers

## Abstract

The rational design of photonic crystal photocatalysts has attracted significant interest in order to improve their light harvesting and photocatalytic performances. In this work, an advanced approach to enhance slow light propagation and visible light photocatalysis is demonstrated for the first time by integrating a planar defect into CoO_x_-TiO_2_ inverse opals. Trilayer photonic crystal films were fabricated through the successive deposition of an inverse opal TiO_2_ underlayer, a thin titania interlayer, and a photonic top layer, whose visible light activation was implemented through surface modification with CoO_x_ nanoscale complexes. Optical measurements showed the formation of “donor”-like localized states within the photonic band gap, which reduced the Bragg reflection and expanded the slow photon spectral range. The optimization of CoO_x_ loading and photonic band gap tuning resulted in a markedly improved photocatalytic performance for salicylic acid degradation and photocurrent generation compared to the additive effects of the constituent monolayers, indicative of light localization in the defect layer. The electrochemical impedance results showed reduced recombination kinetics, corroborating that the introduction of an optical defect into inverse opal photocatalysts provides a versatile and effective strategy for boosting the photonic amplification effects in visible light photocatalysis by evading the constraints imposed by narrow slow photon spectral regions.

## 1. Introduction

Tailoring the morphology of semiconductor photocatalysts in the form of three-dimensional (3D) photonic crystal (PC) architectures has been attracting attention as an advanced approach that may selectively improve light harvesting at frequencies of weak electronic absorption by using slow photon effects, i.e., slow light propagation at the photonic band gap’s (PBG) edges [1,2]. Significant progress has been achieved in slow photon-assisted visible light-activated (VLA) photocatalysis through defect engineering [3,4,5] and mostly the heterostructuring of benchmark wide band gap semiconductor photocatalysts such as TiO_2_ with suitable semiconducting [6,7,8,9,10], plasmonic [11,12,13], and graphene [14,15,16] nanomaterials or by PC fabrication using narrow band gap semiconductors that are intrinsically responsive to visible light [17,18,19,20,21]. In all cases, photonic amplification was based on the spectral matching of the red or blue [22,23] slow photon frequency regions to the weak electronic absorption of the photocatalyst, while avoiding detrimental Bragg reflection losses. PBG/slow photon engineering is commonly implemented by varying the PC periodicity, e.g., the macropore void diameter in the case of inverse opal structures [24,25] and the angle of light incidence [26,27] or, more recently, by the compositional tuning of heterostructured WO_3_/TiO_2_ inverse opal films that provides an additional degree of freedom for the control of the PC lattice parameter beyond that of templating spheres [28].

In addition, PC structures have been utilized as inert photonic supports for photocatalytic layers, acting as dielectric mirrors, whose Bragg reflection is tuned to the catalysts’ absorption edge leading to enhanced light harvesting independently of the photocatalytic process [29,30,31,32]. Moreover, PBG engineering of CoO_x_-TiO_2_ bilayer films consisting of an inverse opal substrate and a benchmark mesoporous titania overlayer showed that PCs can be very effective as both photocatalytic layers and Bragg mirrors in multilayer photonic films [33], while a significantly enhanced performance on photoelectrochemical water splitting was reported for bilayer BiVO_4_-WO_3_ photoanodes deposited upon inverse opal WO_3_ underlayers [34]. Very recently, multi-spectral slow photon tuning to the photocatalyst electronic absorption was demonstrated for bilayers consisting of TiO_2_-BiVO_4_ inverse opals with two distinct PBGs leading to enhanced light trapping for VLA photocatalysis [35]. A Fabry–Pérot-like resonator was previously implemented by Eftekhari et al. [36] using a trilayer stack of 3D TiO_2_ PCs consisting of identical top and bottom inverse opal layers and a middle one with larger pores, whose red and blue PBG edges overlapped with the TiO_2_ absorption edge. The trilayer film enabled light trapping between the top and bottom PC layers, leading to a significantly improved photocatalytic activity under UVA light and corroborating the unique prospects of slow photon management for photocatalytic applications in the absence of visible light activation.

An alternative approach to manipulate slow light propagation in PC structures was previously proposed based on the introduction of planar “defects” that disrupt their periodicity and create localized states within the PBG [37]. Optical defect engineering in PCs has been keenly pursued since the early days of the field of photonics for optoelectronic applications [38,39], while planar defects have been reported for polymeric colloidal opals [40] and SiO_2_ inverse opals [41,42] by the deposition of an intermediate layer with the aim of recovering optical transmission within the forbidden PBG spectral range. Additional slow photon regions can therefore be created around the optical defect state, which reduce the group velocity, similar to the band bending at the edges of the photonic band gap (PBG). However, the incorporation of optical defects into PC photocatalysts to expand the relatively narrow slow light spectral range, which limits photonic amplification effects, has not been explored until now. There have been only a few reports addressing this challenge, primarily focusing on engineering multiple slow photon spectral regions within multilayer PC structures with different lattice constants [35,36].

This work demonstrates for the first time that introducing an optical defect within monolithic CoO_x_-TiO_2_ inverse opals is an effective strategy to enhance visible light harvesting and photocatalytic performance by reducing Bragg reflection losses and expanding the slow photon spectral regions. Composite PC films were fabricated by the successive deposition of an inverse opal TiO_2_ substrate, a thin mesoporous layer of the benchmark Aeroxide P25 titania catalyst acting as planar defect, and a TiO_2_ inverse opal top layer identical to the bottom one. Visible light activation of the trilayer PC films was performed through their surface modification with CoO_x_ “molecular” complexes at various stages of the multilayer deposition in order to optimize visible light absorption. The optimization of CoO_x_ loading in combination with PBG engineering resulted in a greatly improved photocatalytic performance of the CoO_x_-TiO_2_ trilayer PCs, which was evaluated based on salicylic acid (SA) degradation as well as photoelectrochemical measurements under visible light. Marked enhancements of the SA degradation rate and photocurrent density as well as reduced recombination kinetics were observed, indicating the unique potential of optical defect engineering in augmenting the reactivity of PC photocatalysts.

## 2. Materials and Methods

### 2.1. Photonic Film Fabrication

**Figure 1:**
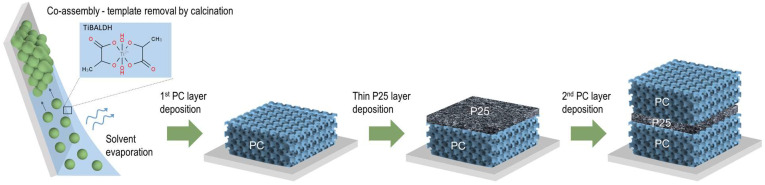
Schematic illustration of the PC/P25/PC trilayer deposition process.

TiO_2_ inverse opal underlayers were deposited on glass substrates in a single-step process via the evaporation-induced co-assembly of 0.1% w/v monodisperse poly(methyl methacrylate) (PMMA) colloidal spheres with a hydrolyzed Ti alkoxide precursor based on a titanium(IV) bis(ammonium lactato)dihydroxide (TiBALDH) 50 wt.% aqueous solution, followed by calcination at 500 °C. PMMA templating spheres with 406 and 499 nm diameters were selected to deposit the photonic substrates, labeled as PC406 and PC499, leading to well-defined PBGs (in water) that approached the spectral region of electronic absorption of CoO_x_ oxides [43]. A thin nanocrystalline TiO_2_ layer of about 200 nm was then deposited by spin coating a paste made from the benchmark Aeroxide® P25 titania catalyst at 2000 rpm for 60 s on the PC substrate, followed by drying at 120 °C and calcination at 450 °C. Finally, a TiO_2_ inverse opal upper layer was deposited on the P25/PC bilayer films under the same conditions as the original substrate, PC406 or PC499, leading to PC406/P25/PC406 and PC499/P25/PC499 trilayer structures, as depicted in Figure 1.

A surface modification using nanoscale Co oxides was performed via the chemisorption of cobalt(II) acetylacetonate dihydrate Co(acac)_2_(H_2_O)_2_ on the titania films after immersion in 100 mL of a 10^−3^ Μ solution (3:17 v/v EtOH/n-hexane) for 24 h followed by thorough washing with the same solvent, drying, and post-calcination in air at 500 °C for 1 h [43]. The CoO_x_ modification was performed using the chemisorption–calcination method at various stages of the films’ deposition to achieve the optimal absorption of visible light. In particular, the trilayer films CoO_x_-PC406/P25/PC406, PC406/CoO_x_-P25/PC406, CoO_x_-PC406/CoO_x_-P25/PC406, and PC499/CoO_x_-P25/PC499 were deposited as well as the reference films CoO_x_-PC406, CoO_x_-PC499, and CoO_x_-P25.

### 2.2. Materials Characterization

The films’ morphology and phase composition were studied using a scanning electron microscope (SEM, Quanta Inspect, FEI, Eindhoven, Netherlands) and a micro-Raman microscope (inVia Reflex, Renishaw, London, UK) with a ×50 objective at 785 nm excitation and a low power density (0.1 mW/μm^2^). The optical properties were investigated by specular and diffuse reflectance spectroscopy on a Cary 60 UV-Vis spectrometer equipped with fiber-optic diffuse reflectance and specular reflectance accessories, using a Halon powder standard and a UV-enhanced Al mirror for baseline measurements, respectively. Photoelectrochemical experiments were performed in a three-electrode configuration on a CS350 potentiostat/galvanostat using the photonic films deposited on cleaned fluorine-doped tin oxide (FTO) transparent conductive glass (7 ohms/sq Pilkington) substrates as working electrodes, Ag/AgCl as a reference electrode, and a Pt foil as a counter electrode in 0.5 M NaOH aqueous electrolyte. The measurements were carried out in the dark and under visible light illumination provided by a 300 W Xe lamp with a 400 nm long-pass filter at 100 mW/cm^2^. Electrochemical impedance spectroscopy (EIS) was carried out at open-circuit voltage (V_OC_) in the frequency range of 10^4^–10^−1^ Hz with an ac amplitude of 10 mV. Mott–Schottky measurements were carried out at 1 kHz and a scan rate of 10 mV/s at each potential.

### 2.3. Photocatalytic Evaluation

The photocatalytic activity of trilayer PC and reference films was evaluated on the aqueous phase degradation of SA under visible light. Films with an area of 2 cm^2^ were placed horizontally to vials containing aqueous SA solutions (3 mL, 25 μΜ), which were stirred for 60 min in the dark to reach adsorption–desorption equilibrium, while adsorption was enhanced by stabilizing the solution’s pH at 3 using diluted HCl. UV–Vis irradiation was provided by a 150 W Xe lamp along with a long-pass edge filter with a 305 nm cut-on and a heat-reflective mirror, while visible light was selected using an additional 400 nm long-pass filter. The horizontal beam was directed onto the film surface by a UV-enhanced Al mirror with an incident power density on the film surface of 76 mW/cm^2^. Aliquots of 0.5 mL were periodically withdrawn from the SA solution and analyzed in a 10 mm quartz micro cell in the Cary 60 spectrophotometer. The photocatalytic experiments were performed in triplicate, and standard errors were calculated for the mean kinetic constants.

## 3. Results and Discussion

### 3.1. Morphological, Structural, and Optical Properties

**Figure 2:**
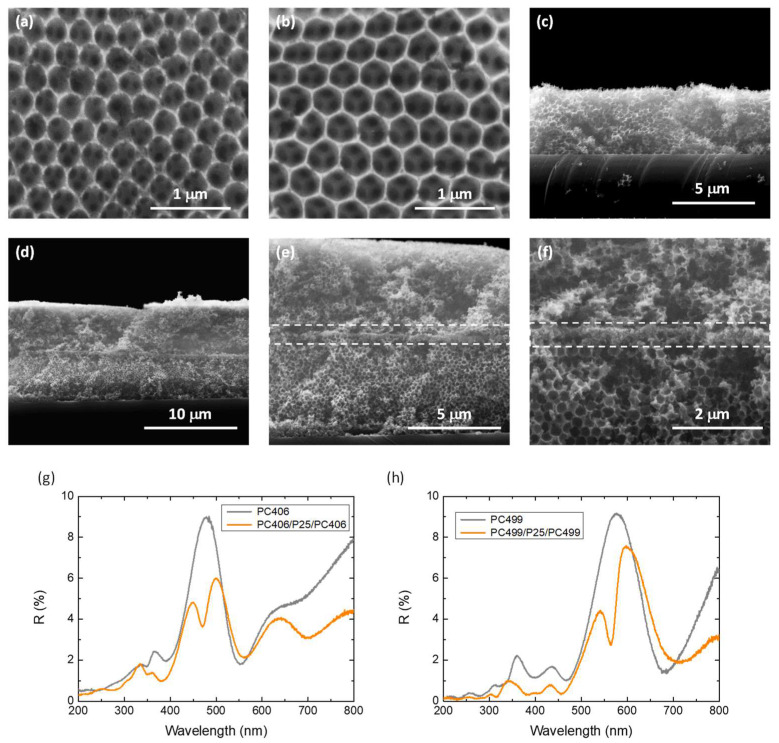
SEM images of the (**a**) PC406 and (**b**,**c**) PC499 inverse opal substrates as well as (**d**–**f**) cross-sections of the PC406/P25/PC406 trilayer photonic films at different magnifications. Specular reflectance (R%) spectra for the (**g**) PC406/P25/PC406 και (**h**) PC499/P25/PC499 in comparison to the monolithic PC406 and PC499 inverse opals.

SEM images of the co-assembled PC406 and PC499 photonic substrates, shown in Figure 2a–c, display the formation of well-organized periodic inverse opal structures with macropore sizes of 255 and 310 nm, respectively. Cross-sectional SEM images of the PC406/CoO_x_-P25/PC406 trilayer films at successively higher magnifications, shown in Figure 2d–f, indicate the formation of a thin nanocrystalline P25 layer of about 200 nm thickness, which is inserted between the two photonic layers interrupting the periodicity of the inverse opal structure. Figure 2g,h show specular reflectance (R%) measurements at a 15° incidence angle for the PC406/P25/PC406 and PC499/P25/PC499 trilayer films in comparison to the corresponding monolithic PC406 and PC499 inverse opals (without an intermediate P25 layer).

The incomplete PBGs (stop bands) of the PC406 and PC499 inverse opals were determined from the corresponding Bragg reflections at 486 and 578 nm, respectively. The stop band positions can be approximated using the modified Bragg’s law for first-order diffraction from the (111) planes of the fcc inverse opal structure [9]

(1) $$\lambda =2d_{111}\sqrt{n_{eff}^{2}-{sin}^{2}\theta }$$

where λ is the stop band wavelength, $d_{111}=\sqrt{2/3}D$ is the spacing between the (111) planes, D is the macropore diameter, and $n_{eff}^{2}=n_{sphere}^{2}f+n_{{TiO}_{2}}^{2}(1-f)$ shows the volume-weighted average of the sphere’s refractive index ${(n}_{sphere})$ and titania $\left(n_{{TiO}_{2}}\right)$, while f is the sphere-filling fraction (f=0.74 for the fcc lattice), and θ is the angle between the incident beam and the [111] direction. Using the experimental PBG wavelengths at θ=15° and the sphere diameters for $n_{{TiO}_{2}}=2.55\;and$ $n_{sphere}=n_{air}=1.0$, the n values and the titania filling fractions $\left(1-f\right)$ were determined in air (Table 1). These values were much smaller than the theoretical one of 0.26 for complete filling of the inverse fcc lattice, supporting the mesoporosity of the nanocrystalline anatase skeleton. Furthermore, using the obtained $\left(1-f\right)$ values for $n_{sphere}=n_{H_{2}O}=1.33$, the stop bands were calculated at 603 and 731 nm for PC406 and PC499 at 0° incidence in water, where the photocatalytic reactions took place.

Importantly, a distinctive local minimum within the Bragg reflection of the monolithic PCs was clearly observed in the corresponding R% spectra for the PC406/P25/PC406 and PC499/P25/PC499 trilayer photonic films (Figure 2g,h). The observed dip within the Bragg R% peak indicates the creation of a localized state that enhances transmittance through the inverse opal PBG, confirming the formation of a structural “defect” that interrupts the periodicity of the PC structure [41]. The observed local minima in the R% spectra of the trilayer photonic films were shifted to lower wavelengths with respect to the Bragg reflection approaching the high-energy PBG edge. Such a spectral shift leads to donor-like optical states similar to the localized electronic states close to the conduction band in n-type semiconductors. This behavior is in close agreement with previous results on high-quality SiO_2_ inverse opals fabricated by chemical vapor deposition, incorporating planar defects of a homogeneous SiO_2_ layer with thicknesses smaller than the lattice constant ($a=\sqrt{2D}$) of the photonic crystal [41,42]. In this case, besides the decrease in group velocity due to the reduced gradients of the dispersion relation in the spectral region of the planar defect likewise the slow photon effects at the PBG edges, a strong localization of light is also expected [38], which can enhance the photocatalytic process through the mesoporous VLA CoO_x_-P25 defect layer.

**Figure 3:**
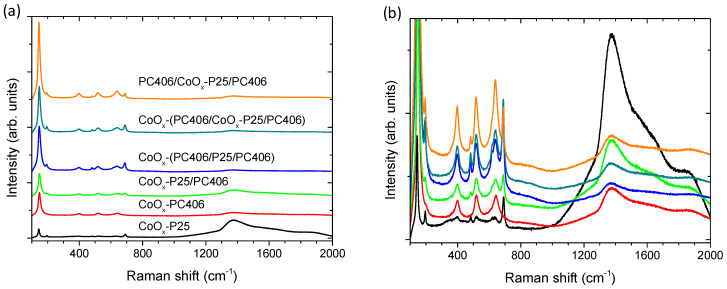
Raman spectra of the trilayer films CoO_x_-PC406/P25/PC406, PC406/CoO_x_-P25/PC406, and CoO_x_-PC406/CoO_x_-P25/PC406 in comparison to CoO_x_-P25/PC406, CoO_x_-PC406 and CoO_x_-P25 reference films at 785 nm and different intensity scales (**a**,**b**). Shaded bands indicate the Co_3_O_4_ Raman modes.

The structural properties and phase composition of the trilayer photonic films were studied by Raman spectroscopy after CoO_x_ surface modification through the Co(acac)_2_(H_2_O)_2_ chemisorption–calcination treatment at various stages of their fabrication in comparison to the CoO_x_-P25/PC406, CoO_x_-PC406, and CoO_x_-P25 reference films (Figure 3). The monolithic TiO_2_ inverse opals showed the characteristic Raman-active phonons of anatase at approximately 147 (E_g_), 197 (E_g_), 398 (B_1g_), 518 (A_1g_ + B_1g_), and 642 cm^−1^ (E_g_), confirming their crystallization in the single anatase phase after calcination at 500 °C. The anatase modes, especially the most intense low-frequency E_g_ band, exhibited appreciable shifts and broadening for the PC films compared to P25, due to the formation of small-size (≤10 nm) anatase nanocrystals in the co-assembled TiO_2_ inverse opals using the TiBALDH precursor that cause the breakdown of the *q* = 0 selection rule for Raman scattering in titania nanomaterials [15]. The Raman spectra of the surface-modified titania films showed, in addition to the anatase TiO_2_ phonons (the weak peaks of the rutile phase are only discernible in the P25 films), characteristic peaks of the Co_3_O_4_ spinel phase at 194, 483, 619, and 692 cm^−1^ [44]. The latter bands were significantly enhanced due to the resonance Raman scattering with excitation at 785 nm, which approaches the strong electronic transition of Co_3_O_4_ at about 750 nm [45]. The Co_3_O_4_ Raman modes were observed in all multilayer films containing the P25 layer, in contrast to the CoO_x_-PC406 films, where these vibrations were not observed, confirming the preferential formation of the Co_3_O_4_ spinel in the mesoporous CoO_x_-P25 films [43]. This was directly observed in the corresponding Raman spectrum of the reference CoO_x_-P25 thin film, where the spinel Raman peaks and the parasitic fluorescence of the glass substrate (1200–1600 cm^−1^) were dominant. The relative intensity of the Co_3_O_4_ Raman peaks with respect to the TiO_2_ Raman mode intensities in the trilayer photonic films depended on the stage of the application of the surface modification.

The lowest intensity was observed for the PC406/CoO_x_-P25/PC406 films, where the surface modification was performed after the deposition of the P25 interlayer in the photonic substrate, while the highest intensity was detected for the films where the CoO_x_ deposition was performed after the completion of the trilayer film fabrication. However, it should be pointed out that, apart from the deposition of nanoscopic Co_3_O_4_, the formation of monovalent oxides CoO and Co_2_O_3_ is also possible, which show weak Raman scattering [44,46] especially in the photonic layers, as previously reported for CoO_x_-TiO_2_ P25/PC bilayer films [33].

**Figure 4:**
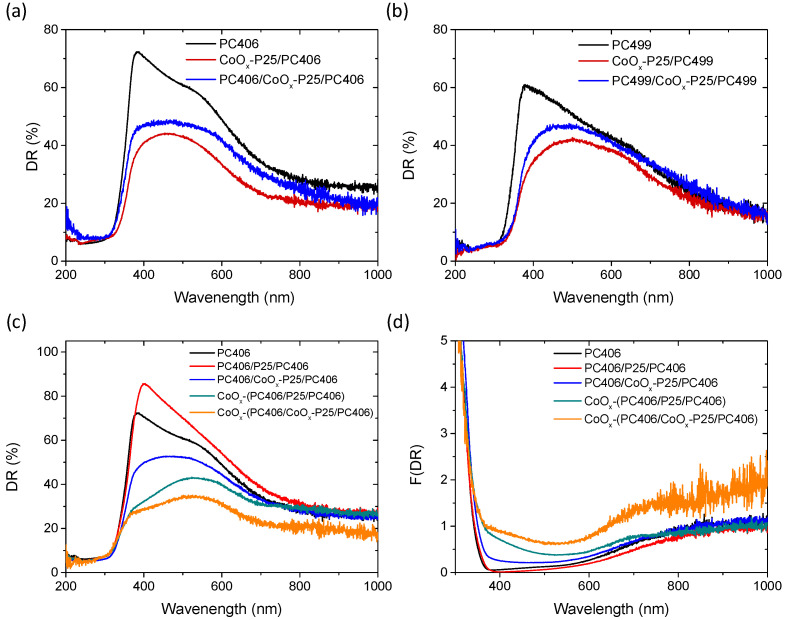
Diffuse (DR%) reflectance spectra for the mono-, bi-, and tri-layer photonic films: (**a**) PC406, CoO_x_-P25/PC406, PC406/CoO_x_-P25/PC406 and (**b**) PC499, CoO_x_-P25/PC499, and PC499/CoO_x_-P25/PC499. (**c**) DR% and (**d**) the corresponding Kubelka–Munk F(DR) absorbance spectra of the PC406/CoO_x_-P25/PC406, CoO_x_-PC406/P25/PC406, and CoO_x_-PC406/CoOx-P25/PC406 trilayer films in comparison to the PC406 and PC406/P25/PC406 reference ones.

The influence of CoO_x_ surface modification on the optical properties of the trilayer films was observed in the diffuse reflectance (DR%) spectra, which are presented in Figure 4. The deposition of CoO_x_ by a chemisorption–thermal treatment cycle of the Co(acac)_2_(H_2_O)_2_ complex following the deposition of the P25 mesoporous layer on the PC406 and PC499 photonic substrates resulted in a significant reduction in DR% over a wide spectral range due to the formation of CoO_x_ oxides, as shown in Figure 4a,b. The deposition of the TiO_2_ photonic top layer on the P25/PC bilayer films to form the trilayer structure resulted in an increase in DR%, which, however, remained lower than that of the original PC substrate. Surface modification of the trilayer films with CoO_x_ caused an even greater decrease in DR% due to the formation of CoO_x_ in the upper photonic layer, which became particularly pronounced in the case of CoO_x_-PC406/CoO_x_-P25/PC406 films, as shown in Figure 4c, where CoO_x_ was deposited twice. 

In all cases, the drop in the DR% after CoO_x_ modification occurred mainly in the 400-500 nm spectral range and to a lesser extent at wavelengths above 700 nm, which were also detected in the corresponding Kubelka–Munk absorbance spectra (Figure 4d). These absorption bands comply favorably with the electronic transitions at about 2.8 and 1.7 eV for Co_3_O_4_ [47], corroborating the preferential spinel formation detected by Raman spectroscopy on the P25 interlayer (Figure 3) as well as the single-valence CoO and Co_2_O_3_ oxides presenting wider band gaps of approximately 2.6 and 1.8 eV, respectively [48].

### 3.2. Photocatalytic Activity

**Figure 5:**
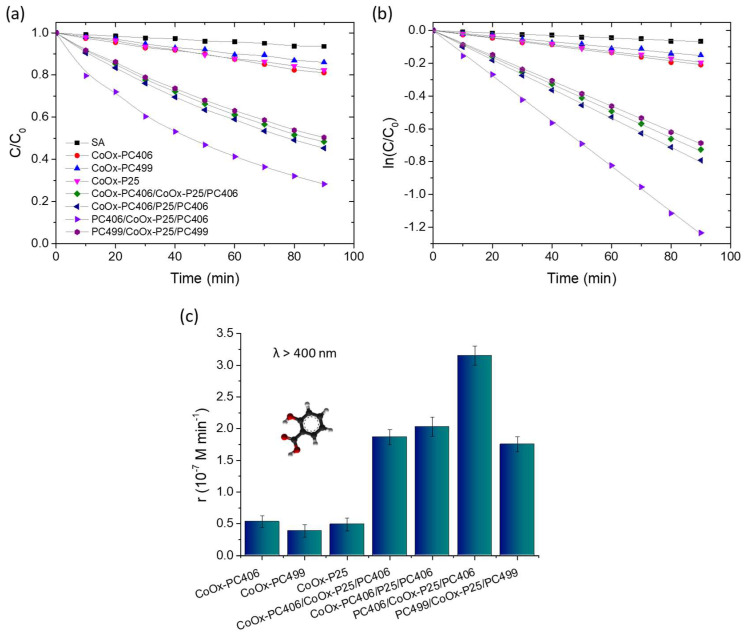
(**a**,**b**) SA photodegradation kinetics and (**c**) reaction rates for the CoO_x_-surface modified trilayer photonic films in comparison to the constituent monolithic CoO_x_-TiO_2_ films under visible light (λ > 400 nm) irradiation.

The photocatalytic performance of the trilayer photonic films compared to their constituent monolithic layers was evaluated in the photodegradation of SA under visible light irradiation (λ > 400 nm), using aqueous solutions with pH = 3 that favor the chemisorption of SA on TiO_2_ and its direct oxidation via photoinduced holes [16]. All the trilayer films showed a strong photocatalytic activity in the degradation of SA, whose concentration (*C*) was determined from the absorption peak at 300 nm, which decreased gradually with irradiation time, as shown in Figure 5a. In all cases, the variation in ln(*C*/*C*_0_) with time *t* presented a linear dependence, where *C*_0_ was the initial concentration of SA at *t* = 0 after its adsorption on the films’ surface in the dark for 30 min, showing that the photocatalytic reaction follows first-order kinetics (Figure 5b). Figure 5c shows the change in the reaction rates *r* = *kC*_0_, which were determined from the kinetic constants *k* obtained from the slopes of the linear plots ln(*C*/*C*_0_) vs *t* and the corresponding *C*_0_ values. The surface-modified PC406/P25/PC406 films presented significantly higher reaction rates than the sum of the respective rates of the constituent reference CoO_x_-P25 and CoO_x_-PC406 films, regardless of the processing step in which the CoO_x_ deposition was carried out.

This behavior indicates that the trilayer structure can enhance visible light harvesting by extending the slow light spectral region for the CoO_x_-PC406 inverse opals, which exhibit the best photocatalytic performance in SA decomposition as the two edges, high and low-energy, of the PBG expected at ~600 nm in water (Table 1) overlap with the electronic absorption bands of CoO_x_-TiO_2_. It is noteworthy that, among the surface-modified trilayer films, the PC406/CoO_x_-P25/PC406 films exhibited the best reaction rate even though they presented the lowest visible light optical absorption (Figure 4c) as the upper PC406 layer is not optically active in the visible range due to the deposition of CoO_x_ in the intermediate fabrication stage of the trilayer structure, i.e., on the P25/PC406 bilayer film. This behavior shows that the enhancement of visible light collection and photocatalytic activity in the trilayer films is largely caused by the localization of light in the CoO_x_-P25 interlayer, which, despite its small thickness, exhibits a high photocatalytic activity. In addition, the strong optical absorption due to the higher CoO_x_ concentration in the CoO_x_-PC406/P25/PC406 and CoO_x_-PC406/CoO_x_-P25/PC406 films can deteriorate their photonic properties as well as the light transmittance in the trilayer structure. An appreciable photocatalytic performance was also observed for the PC499/CoO_x_-P25/PC499 trilayer films, where visible light localization in the PBG region of PC499 in water is expected at ~730 nm (Table 1), which overlaps with the electronic absorption region of CoO_x_ oxides and particularly the nanoscale Co_3_O_4_ clusters formed in the P25 mesoporous interlayer that absorb strongly at 450 and 750 nm [45,47]. This indicates that Bragg reflection losses in the PC499 layers and also the contribution of the most active CoO_x_-PC406 photonic substrate are important to the overall photocatalytic performance.

It should be noted that the introduction of a mesoporous P25 nanocrystalline interlayer with a surface area of about 40 m^2^ g^−1^ [49] is not expected to appreciably influence the pore structure of the monolithic PC406 and PC499 inverse opal films with surface areas of about 64 and 68 m^2^ g^−1^, respectively, arising mainly from the wall mesoporosity of the co-assembled inverse opal films [43] and, thus, the pore structure of the trilayer films. Although there are no reports in the literature on the performance of PC photocatalysts with optical defects, a general comparison can be made by examining the enhancement factor (EF) of the observed reaction rates in water pollutant degradation, relative to those of bilayer PCs, where amplification is pursued by Bragg reflection and multi-spectral slow photons via double PBG formation [33,35]. The EF, calculated from the ratio of the reaction rate for the best-performing PC406/CoO_x_-P25/PC406 film to the sum of the *r* values for the individual CoO_x_-PC406 support, the CoO_x_-P25 interlayer, and the bare PC406 top layer, reached 2.67. This is comparable to, yet slightly higher than, the EF of 2.45 obtained for the CoO_x_-P25/PC406 bilayer film in the VLA SA degradation with a thicker P25 top layer [33], and the EF of 2.2 recently reported from the photocatalytic degradation of the rhodamine B dye using BiVO_4_-decorated TiO_2_ bilayer inverse opals compared to the corresponding monolayer PC films [35]. These results suggest that introducing an optical defect through the insertion of a mesoporous layer in inverse opal photocatalysts offers a versatile and competent strategy for enhancing visible light harvesting through slow photons in spectral regions of the weak electronic absorption.

### 3.3. Charge Separation

**Figure 6:**
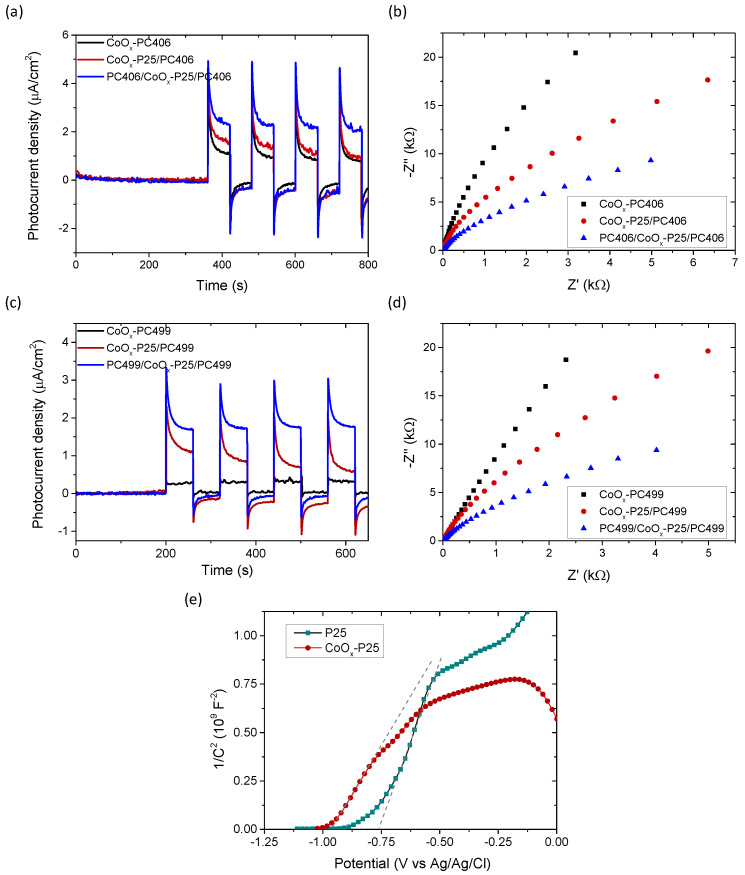
Transient photocurrent density and EIS Nyquist plots in 0.5 Μ NaOH electrolyte under visible light illumination for the mono-, bi-, and tri-layer films (**a**), (**b**) CoO_x_-PC406, CoO_x_-P25/PC406, PC406/CoO_x_-P25/PC406, and (**c**), (**d**) CoO_x_-PC499, CoO_x_-P25/PC499, PC499/CoO_x_-P25/PC499. (**e**) Mott–Schottky plots for the P25 και CoO_x_-P25 films at 1000 Hz.

The photocatalytic responses of PC406/CoO_x_-P25/PC406 and PC499/CoO_x_-P25/PC499 were further studied through photoelectrochemical measurements on films deposited on conductive FTO substrates. Figure 6 shows the photocurrent density produced during photocatalytic water splitting in the presence of mono-, bi-, and tri-layer films as a function of time under chopped visible light illumination (>420 nm) in a 0.5 M NaOH electrolyte at 0.2 V vs. Ag/AgCl as well as the Nyquist plots of the electrochemical impedance under visible light. Upon illumination, the photocurrent density increased instantly to the highest value that corresponds to the current density of photogenerated holes reaching the semiconductor–electrolyte interface. Subsequently, due to surface electron–hole recombination, the photocurrent density decreased exponentially to a steady-state value, which corresponded to the flux of the holes in the electrolyte without undergoing recombination with electrons at the surface [50]. The negative photocurrent overshoot feature that appears when switching the light off has been associated with the back flow of electrons that recombine with the remaining holes at the surface. An increase in both instantaneous (hole current) and steady-state photocurrent density values was observed for the trilayer photonic films, with a higher value in the case of PC406/CoO_x_-P25/PC406 confirming the enhanced visible light harvesting and the improved charge separation for the specific inverse opal diameter in the trilayer structure. The same conclusion is obtained from the corresponding Nyquist diagrams, which show that the charge transfer resistance at the interface of the photoelectrode with the liquid electrolyte, which corresponds to the curvature of the graph of the imaginary part of the impedance –Ζ’’ with respect to the real Z’, is significantly reduced in the trilayer films, regardless of the inverse opal diameter. 

In addition, capacitance measurements $\left(C=-1/{Ζ}^{\prime \prime }\omega \right)$ were performed as a function of the applied potential V at a constant frequency of 1000 Hz for the defect P25 interlayer (Figure 6e). Neglecting the contribution of the Helmholtz layer ${(C}_{H})$, the inverse capacitance at the semiconductor–electrolyte interface for n-type semiconductors such as TiO_2_ is approximated using the inverse capacitance (${1/C}_{sc}\gg {1/C}_{H}$) in the space charge region (depletion zone) according to the following relation

(2) $$\frac{1}{C^{2}}={\left(\frac{1}{C_{sc}}+\frac{1}{C_{H}}\right)}^{2}\approx \frac{1}{{C_{sc}}^{2}}=\frac{2}{eA^{2}\varepsilon \varepsilon_{0}N_{D}}\left(V-V_{fb}-\frac{kT}{e}\right)$$

where *e* is the electron charge, *ε* is the dielectric constant, *A* is the geometric surface of the semiconductor electrode, $\varepsilon_{0}$ is the vacuum permeability, and *k* is Boltzmann constant. *N*_D_ is the concentration of donors of the semiconductor and *V*_fb_ is the flatband potential, which is defined as the potential that neutralizes the bending of the semiconductor energy bands when equilibrium is reached at the semiconductor–electrolyte interface through the equation of the Fermi energy with the electrochemical potential of the electrolyte.

The linear part of the plot of $1/C^{2}\mathrm{vs}.\;V$ (Mott–Schottky plot) with the applied potential was used to determine *N*_D_ and *V*_fb_ from the slope and the intercept with the potential axis [51]. Figure 6e shows the Mott-Schottky diagrams of the P25 and CoO_x_-P25 films, which form the intermediate photocatalytic layer (planar defect) for the localization of visible light in the trilayer structure. A shift in *V*_fb_ to negative potentials from −0.78 to −0.99 V vs. Ag/AgCl was observed as well as an increase in the donor concentration from 4.1 × 10^19^ cm^−3^ to 7.2 × 10^19^ cm^−3^ in the CoO_x_-P25 film compared to the unmodified mesoporous titania P25 film. This behavior can be attributed to the formation of a surface band in the CoO_x_/TiO_2_ composite system that shifts the valence band of TiO_2_ to lower potentials [52,53] as well as the formation of p-n Co_3_O_4_-TiO_2_ heterojunctions through the preferential formation of Co_3_O_4_ spinel nanoparticles, a p-type semiconductor, on the surface of the modified P25 films [54].

## 4. Conclusions

An alternative structural approach to enhance visible light harvesting and the photocatalytic performance of PC photocatalysts has been implemented for the first time by the introduction of a planar defect in visible light activated CoO_x_-TiO_2_ inverse opal films in order to alleviate the major limitation of slow light propagation within narrow spectral ranges at the PBG edges. The deposition of a mesoporous titania layer within monolithic inverse opals was studied as a means to interrupt the PC periodicity and create localized defect states within the PBG. To this end, trilayer photonic films were fabricated by the successive deposition of an inverse opal TiO_2_ underlayer, a thin mesoporous titania interlayer, and a photonic top layer identical to the original one. Visible light activation of the trilayer photonic films was carried out by their surface modification with low-concentration CoO_x_ “molecular” complexes at various stages of the film fabrication in order to optimize visible light absorption. Optical measurements showed the formation of “donor”-like states within the trilayer PBG, which reduced the Bragg reflection and broadened the slow light spectral regions. The optimization of CoO_x_ loading in combination with PBG engineering resulted in an improved photocatalytic performance of the CoO_x_-TiO_2_ trilayer PCs that were evaluated using SA degradation and photoelectrochemical measurements under visible light. Significant enhancements in the SA degradation rate and photocurrent density along with reduced electron–hole recombination were observed for the optimal trilayer PCs in comparison to the additive effect of the constituent layers and trilayers with variable CoO_x_ loading, indicative of light localization in the middle defect layer. Optical defect engineering within the PBG of PC photocatalysts is, accordingly, concluded to be a promising strategy to enhance slow photon amplification effects and reduce Bragg reflection losses, which can be further combined with suitable compositional modifications for visible light photocatalysis.

## Figures and Tables

**Table 1:** Structural and optical parameters of the TiO_2_ PC substrates.

Film	D (nm)	λ (15°) (nm)	$n_{eff}$ (air)	1−f	$n_{eff}$ (H_2_O)	λ (0°) (nm) (air)	λ (0°) (nm) (H_2_O)
PC406	255(5)	478	1.18(2)	0.070(9)	1.45(2)	490(13)	603(13)
PC499	310(5)	578	1.17(2)	0.067(9)	1.44(2)	593(16)	731(16)

## Data Availability

The original contributions presented in this study are included in the article. Further inquiries can be directed to the corresponding author.

## References

[1] Chen J.I.L., von Freymann G., Choi S.Y., Kitaev V., Ozin G.A. (2006). Amplified photochemistry with slow photons. Adv. Mater..

[2] Curti M., Schneider J., Bahnemann D.W., Mendive C.B. (2015). Inverse opal photonic crystals as a strategy to improve photocatalysis: Underexplored questions. J. Phys. Chem. Lett..

[3] Cai J.M., Wu M.Q., Wang Y.T., Zhang H., Meng M., Tian Y., Li X.A., Zhang J., Zheng L.R., Gong J.L. (2017). Synergetic enhancement of light harvesting and charge separation over surface-disorder engineered TiO2 photonic crystals. Chem.

[4] Wang Y., Peng C., Jiang T., Zhang J., Jiang Z., Li X. (2020). Construction of defect-engineered three-dimensionally ordered macroporous WO_3_ for efficient photocatalytic water oxidation reaction. J. Mater. Chem. A.

[5] Feng L., Wang F., Luo H., Xu Z., Zhao T., Zhu J., Qin Y. (2023). Thermal vacuum de-oxygen fabrication of new catalytic pigments: SiO_2_@TiO_2−x_ amorphous photonic crystals for formaldehyde removal. J. Mater. Chem. B.

[6] Zhao H., Li C.-F., Hu Z.-Y., Liu J., Li Y., Hu J., Van Tendeloo G., Chen L.-H., Su B.-L. (2021). Size effect of bifunctional gold in hierarchical titanium oxide-gold-cadmium sulfide with slow photon effect for unprecedented visible-light hydrogen production. J. Colloid Interface Sci..

[7] Pylarinou M., Toumazatou A., Sakellis E., Xenogiannopoulou E., Gardelis S., Boukos N., Dimoulas A., Likodimos V. (2021). Visible light trapping against charge recombination in FeO_x_–TiO_2_ photonic crystal photocatalysts. Materials.

[8] Madanu T.L., Mouchet S.R., Deparis O., Liu J., Li Y., Su B.-L. (2022). Tuning and transferring slow photons from TiO_2_ photonic crystals to BiVO_4_ nanoparticles for unprecedented visible light photocatalysis. J. Colloid Interface Sci..

[9] Loukopoulos S., Sakellis E., Kostakis M.G., Gerokonstantis D.-T., Tsipas P., Gardelis S., Kontos A.G., Katsaros F.K., Sideratou Z., Romanos G.E. (2023). Co-assembled MoS_2_–TiO_2_ inverse opal photocatalysts for visible light-activated pharmaceutical photodegradation. ACS Omega.

[10] Hedrich C., James N.T., Maragno L.G., De Lima V., González S.Y.G., Blick R.H., Zierold R., Furlan K.P. (2024). Enhanced photocatalytic properties and photoinduced crystallization of TiO_2_–Fe_2_O_3_ inverse opals fabricated by atomic layer deposition. ACS Appl. Mater. Interfaces.

[11] Collins G., Lonergan A., McNulty D., Glynn C., Buckley D., Hu C., O’Dwyer C. (2020). Semiconducting metal oxide photonic crystal plasmonic photocatalysts. Adv. Mater. Interfaces.

[12] Raja-Mogan T., Lehoux A., Takashima M., Kowalska E., Ohtani B. (2021). Slow photon-induced enhancement of photocatalytic activity of gold nanoparticle-incorporated titania inverse opal. Chem. Lett..

[13] Temerov F., Pham K., Juuti P., Mäkelä J.M., Grachova E.V., Kumar S., Eslava S., Saarinen J.J. (2020). Silver-decorated TiO_2_ inverse opal structure for visible light-induced photocatalytic degradation of organic pollutants and hydrogen evolution. ACS Appl. Mater. Interfaces.

[14] Boppella R., Kochuveedu S.T., Kim H., Jeong M.J., Mota F.M., Park J.H., Kim D.H. (2017). Plasmon-sensitized graphene/TiO_2_ inverse opal nanostructures with enhanced charge collection efficiency for water splitting. ACS Appl. Mater. Interfaces.

[15] Diamantopoulou A., Sakellis E., Gardelis S., Tsoutsou D., Glenis S., Boukos N., Dimoulas A., Likodimos V. (2019). Advanced photocatalysts based on reduced nanographene oxide–TiO_2_ photonic crystal films. Materials.

[16] Apostolaki M.-A., Toumazatou A., Antoniadou M., Sakellis E., Xenogiannopoulou E., Gardelis S., Boukos N., Falaras P., Dimoulas A., Likodimos V. (2020). Graphene quantum dot-TiO_2_ photonic crystal films for photocatalytic applications. Nanomaterials.

[17] Oh Y., Yang W., Tan J., Lee H., Park J., Moon J. (2019). Boosting visible light harvesting in p-type ternary oxides for solar-to-hydrogen conversion using inverse opal structure. Adv. Funct. Mater..

[18] Zhu H., Zhang Y., Zhu J., Li Y., Jiang S., Wu N., Wei Y., Zhou J., Song Y. (2020). Crack-free hematite inverse opal photo-anodes for enhancing photo-electrochemical water splitting. J. Mater. Chem. A.

[19] Lin Q., Liang S., Wang J., Zhang R., Wang X. (2022). Cadmium sulfide 3D photonic crystal with hierarchically ordered macropores for highly efficient photocatalytic hydrogen generation. Inorg. Chem..

[20] Pylarinou M., Sakellis E., Tsipas P., Romanos G.E., Gardelis S., Dimoulas A., Likodimos V. (2023). Mo-BiVO_4_/Ca-BiVO_4_ homojunction nanostructure-based inverse opals for photoelectrocatalytic pharmaceutical degradation under visible light. ACS Appl. Nano Mater..

[21] Dong Y., Ai F., Sun-Waterhouse D., Murai K.-I., Moriga T., Waterhouse G.I.N. (2023). Optical and photocatalytic properties of three-dimensionally ordered macroporous Ta_2_O_5_ and Ta_3_N_5_ inverse opals. Chem. Mater..

[22] Deparis O., Mouchet S.R., Su B.-L. (2015). Light harvesting in photonic crystals revisited: Why do slow photons at the blue edge enhance absorption?. PCCP.

[23] Zhang J., Cai X., Fu X., Teng D., Murtaza G., Meng Z., Jia Z., Qiu L. (2024). Slow light effect enhances the photocatalytic effect of inverse opal TiO_2_-based photonic nanocrystals. ACS Appl. Nano Mater..

[24] Liu J., Jin J., Li Y., Huang H.-W., Wang C., Wu M., Chen L.-H., Su B.-L. (2014). Tracing the slow photon effect in a ZnO inverse opal film for photocatalytic activity enhancement. J. Mater. Chem. A.

[25] Curti M., Mendive C.B., Grela M.A., Bahnemann D.W. (2017). Stopband tuning of TiO_2_ inverse opals for slow photon absorption. Mater. Res. Bull..

[26] Curti M., Zvitco G., Grela M.A., Mendive C.B. (2018). Angle dependence in slow photon photocatalysis using TiO_2_ inverse opals. Chem. Phys..

[27] Wang L., Mogan T.R., Wang K., Takashima M., Ohtani B., Kowalska E. (2022). Fabrication and characterization of inverse-opal titania films for enhancement of photocatalytic activity. ChemEngineering.

[28] Apostolaki M.-A., Sakellis E., Tsipas P., Giannouri M., Gardelis S., Boukos N., Dimoulas A., Likodimos V. (2022). Three-phase co-assembly of compositionally tunable WO_3_/TiO_2_ inverse opal photoelectrodes. Appl. Surf. Sci..

[29] Li P., Chen S.-L., Wang A.-J., Wang Y. (2015). Probing photon localization effect between titania and photonic crystals on enhanced photocatalytic activity of titania film. Chem. Eng. J..

[30] Zhang R., Zeng F., Pang F., Ge J. (2018). Substantial enhancement toward the photocatalytic activity of CdS quantum dots by photonic crystal-supporting films. ACS Appl. Mater. Interfaces.

[31] Xie C., Fan T., Wang A., Chen S.-L. (2018). Enhanced visible-light photocatalytic activity of a TiO_2_ membrane-assisted with n-doped carbon quantum dots and SiO_2_ opal photonic crystal. Ind. Eng. Chem. Res..

[32] Lan D., Sheng W., Fu Q., Ge J. (2023). Enhancement of CO_2_ photoreduction efficiency by supporting blue TiO_2_ with photonic crystal substrate. Nano Res..

[33] Loukopoulos S., Toumazatou A., Sakellis E., Xenogiannopoulou E., Boukos N., Dimoulas A., Likodimos V. (2020). Heterostructured CoO_x_–TiO_2_ mesoporous/photonic crystal bilayer films for enhanced visible-light harvesting and photocatalysis. Materials.

[34] Taga Y., Pan Z., Katayama K., Sohn W.Y. (2022). BiVO_4_-Dotted WO_3_ Photoanode with an inverse opal underlayer for photoelectrochemical water splitting. ACS Appl. Energy Mater..

[35] Madanu T.L., Chaabane L., Mouchet S.R., Deparis O., Su B.-L. (2023). Manipulating multi-spectral slow photons in bilayer inverse opal TiO_2_@BiVO_4_ composites for highly enhanced visible light photocatalysis. J. Colloid Interface Sci..

[36] Eftekhari E., Broisson P., Aravindakshan N., Wu Z., Cole I.S., Li X., Zhao D., Li Q. (2017). Sandwich-structured TiO_2_ inverse opal circulates slow photons for tremendous improvement in solar energy conversion efficiency. J. Mater. Chem. A.

[37] Galisteo-López J.F., Galli M., Andreani L.C., Mihi A., Pozas R., Ocaña M., Míguez H. (2007). Phase delay and group velocity determination at a planar defect state in three dimensional photonic crystals. Appl. Phys. Lett..

[38] Yablonovitch E., Gmitter T.J., Meade R.D., Rappe A.M., Brommer K.D., Joannopoulos J.D. (1991). Donor and acceptor modes in photonic band structure. Phys. Rev. Lett..

[39] Karathanos V., Modinos A., Stefanou N. (1994). Planar defects in photonic crystals. J. Condens. Matter Phys..

[40] Pozas R., Mihi A., Ocaña M., Míguez H. (2006). Building nanocrystalline planar defects within self-assembled photonic crystals by spin-coating. Adv. Mater.

[41] Palacios-Lidón E., Galisteo-López J.F., Juárez B.H., López C. (2004). Engineered planar defects embedded in opals. Adv. Mater.

[42] Tétreault N., Mihi A., Míguez H., Rodríguez I., Ozin G.A., Meseguer F., Kitaev V. (2004). Dielectric planar defects in colloidal photonic crystal films. Adv. Mater.

[43] Toumazatou A., Antoniadou M., Sakellis E., Tsoutsou D., Gardelis S., Romanos G.E., Ioannidis N., Boukos N., Dimoulas A., Falaras P. (2020). Boosting visible light harvesting and charge separation in surface modified TiO_2_ photonic crystal catalysts with CoO_x_ nanoclusters. Mater. Adv..

[44] Rivas-Murias B., Salgueiriño V. (2017). Thermodynamic CoO–Co_3_O_4_ crossover using Raman spectroscopy in magnetic octahedron-shaped nanocrystals. J. Raman Spectrosc..

[45] Qiao L., Xiao H.Y., Meyer H.M., Sun J.N., Rouleau C.M., Puretzky A.A., Geohegan D.B., Ivanov I.N., Yoon M., Weber W.J. (2013). Nature of the band gap and origin of the electro-/photo-activity of Co_3_O_4_. J. Mater. Chem. C.

[46] Li Y., Qiu W., Qin F., Fang H., Hadjiev V.G., Litvinov D., Bao J. (2016). Identification of cobalt oxides with Raman scattering and Fourier Transform infrared spectroscopy. J. Phys. Chem. C.

[47] Singh V., Kosa M., Majhi K., Major D.T. (2014). Putting DFT to the test: A first-principles study of electronic, magnetic, and optical properties of CO_3_O_4_. J. Chem. Theory Comput..

[48] Singh V., Major D.T. (2016). Electronic structure and bonding in Co-based single and mixed valence oxides: A quantum chemical perspective. Inorg. Chem..

[49] Kontos A.I., Kontos A.G., Tsoukleris D.S., Bernard M.-C., Spyrellis N., Falaras P. (2008). Nanostructured TiO_2_ films for DSSCS prepared by combining doctor-blade and sol–gel techniques. J. Mater. Process. Technol..

[50] Dunn H.K., Feckl J.M., Müller A., Fattakhova-Rohlfing D., Morehead S.G., Roos J., Peter L.M., Scheu C., Bein T. (2014). Tin doping speeds up hole transfer during light-driven water oxidation at hematite photoanodes. PCCP.

[51] Hankin A., Bedoya-Lora F.E., Alexander J.C., Regoutz A., Kelsall G.H. (2019). Flat band potential determination: Avoiding the pitfalls. J. Mater. Chem. A.

[52] Jin Q., Yamamoto H., Yamamoto K., Fujishima M., Tada H. (2013). Simultaneous induction of high level thermal and visible-light catalytic activities to titanium (IV oxide by surface modification with cobalt(III) oxide clusters. PCCP.

[53] Tada H., Jin Q., Iwaszuk A., Nolan M. (2014). Molecular-scale transition metal oxide nanocluster surface-modified titanium dioxide as solar-activated environmental catalysts. J. Phys. Chem. C.

[54] Lang D., Cheng F., Xiang Q. (2016). Enhancement of photocatalytic H_2_ production activity of CdS nanorods by cobalt-based cocatalyst modification. Catal. Sci. Technol..

